# Involvement of formyl peptide receptors in receptor for advanced glycation end products (RAGE) - and amyloid beta 1-42-induced signal transduction in glial cells

**DOI:** 10.1186/1750-1326-7-55

**Published:** 2012-11-20

**Authors:** Alexander Slowik, Julika Merres, Anne Elfgen, Sandra Jansen, Fabian Mohr, Christoph J Wruck, Thomas Pufe, Lars-Ove Brandenburg

**Affiliations:** 1 Department of Anatomy and Cell Biology, RWTH Aachen University, Wendlingweg 2, 52074 Aachen, Germany; 2 Department of Neuroanatomy, RWTH Aachen University, Aachen, Germany

**Keywords:** Alzheimer disease, Amyloid beta 1–42, S100B, Formyl peptide receptor, Glial cell, Astrocytes, Microglia, RAGE, Signal transduction

## Abstract

**Background:**

Recent studies suggest that the chemotactic G-protein-coupled-receptor (GPCR) formyl-peptide-receptor-like-1 (FPRL1) and the receptor-for-advanced-glycation-end-products (RAGE) play an important role in the inflammatory response involved in neurodegenerative disorders such as Alzheimer’s disease (AD).

Therefore, the expression and co-localisation of mouse formyl peptide receptor (mFPR) 1 and 2 as well as RAGE in an APP/PS1 transgenic mouse model using immunofluorescence and real-time RT-PCR were analysed. The involvement of rat or human FPR1/FPRL1 (corresponds to mFPR1/2) and RAGE in amyloid-β 1–42 (Aβ1-42)-induced signalling were investigated by extracellular signal regulated kinase 1/2 (ERK1/2) phosphorylation. Furthermore, the cAMP level in primary rat glial cells (microglia and astrocytes) and transfected HEK 293 cells was measured. Formyl peptide receptors and RAGE were inhibited by a small synthetic antagonist WRW4 and an inactive receptor variant delta-RAGE, lacking the intracytoplasmatic domains.

**Results:**

We demonstrated a strong increase of mFPR1/2 and RAGE expression in the cortex and hippocampus of APP/PS1 transgenic mice co-localised to the glial cells. In addition, the Aβ1-42-induced signal transduction is dependant on FPRL1, but also on FPR1. For the first time, we have shown a functional interaction between FPRL1/FPR1 and RAGE in RAGE ligands S100B- or AGE-mediated signalling by ERK1/2 phosphorylation and cAMP level measurement. In addition a possible physical interaction between FPRL1 as well as FPR1 and RAGE was shown with co-immunoprecipitation and fluorescence microscopy.

**Conclusions:**

The results suggest that both formyl peptide receptors play an essential role in Aβ1-42-induced signal transduction in glial cells. The interaction with RAGE could explain the broad ligand spectrum of formyl peptide receptors and their important role for inflammation and the host defence against infections.

## Background

Alzheimer’s disease (AD) is a neurodegenerative disorder characterised by senile plaques and neurofibrillary tangles. An important component of the plaques in the human brain is amyloid-β 1–42 (Aβ1-42), a 42 amino acid peptide fragment derived from sequential proteolytic cleavage of the amyloid precursor protein by beta- and gamma-secretases [1]. Aβ1-42 plays a central role in mediating neurotoxicity and activates glial cells (astrocytes as well as microglia). Elevated levels of non-fibrillar [2,3] and fibrillar Aβ1–42 [4,5] lead to the release of proinflammatory cytokines by activated glial cells. This may subsequently lead to gliosis and cytotoxicity in neurons [6-8]. However, the role of glial cells in the formation of amyloid plaques in Alzheimer’s disease remains unknown [9,10]. The underlying pathogenic mechanisms are not well understood, especially regarding the initial steps of cellular Aβ1-42 uptake and the induction of signal transduction and the consequence for the development of the disease. Recent studies suggest that the chemotactic G-protein-coupled receptor, formyl-peptide-receptor-like 1 (FPRL1), is involved in Aβ1-42 and PrP_106-126_-induced activation and the internalisation in glial cells [11-13]. Furthermore it is indicated that the FPRL1 is expressed on astrocytes and microglia and plays an essential role in the inflammatory response [14].

However, it should be noted that a variety of further receptors were discussed to participate in Aβ1-42-induced glial cell activation and internalisation. In fact, previous results suggest an involvement of the scavenger receptor MARCO (macrophage receptor with collagenous structure) [15,16] and the receptor for advanced glycation endproducts (RAGE) [17]. MARCO is a membrane glycoprotein that can bind to chemically modified low-density lipoproteins or Gram-positive and Gram negative bacteria [18,19]. For MARCO, our recent work shows no involvement in Aβ1-42-induced glial cell activation [11]. However, we are able to show a physical and functional interaction between FPRL1 and MARCO in MARCO ligand fucoidan-induced signaling and in the host defense against brain infections [11,14]. RAGE is a multiligand receptor belonging to the immunoglobulin superfamily [20].

In this study we analysed the expression of formyl peptide receptors and RAGE and their glial localisation using fluorescence microscopy and real-time RT-PCR in an APP/PS1 transgenic mouse model. The murine FPR gene family has at least six members in contrast to only three in humans. *Fpr1* encodes for the murine FPR1 (mFPR1), which is considered to be the murine orthologue of human FPR1, whereas *Fpr-rs2* (mFPR2) encodes for receptors that are similar to the human formyl peptide receptor like 1 (FPRL1) [21]. Furthermore, we examined the involvement of FPRL1, FPR1 and RAGE in Aβ1–42-induced signalling by measured the extracellular-signal regulated kinase 1/2 (ERK 1/2) phosphorylation and cAMP levels in rat glial and transfected HEK293 cells. Also, the involvements of the RAGE receptor ligands S100B as well as AGE-induced signalling were examined. In addition, a functional and physical interaction between FPR1, FPRL1 and RAGE using co-immunoprecipitation and ERK1/2 phosphorylation and cAMP level measurement in rat glial and transfected HEK293 cells was determined. Furthermore, we analysed and quantified the co-localisation between different receptors and S100B or Aβ1–42 in transfected HEK293 cells using fluorescence microscopy. The results suggest that FPRL1 as well as FPR1 play an essential role in Aβ1–42-induced signal transduction in glial cells, and also show the capability of formyl peptide receptors to expand its ligand spectrum by interacting with the RAGE receptor.

## Methods

### Reagents

Human Aβ1–42 and formyl-peptide-receptor antagonist WRW4 [22] were purchased from Dr. P. Henklein (Charité, Berlin, Germany). Peptides were dissolved at 1 and 10 mM concentration in dimethylsulfoxide (DMSO), and Aβ1–42 is present in the soluble form. DMSO used as vehicle in a concentration of 0.1% showed no significant effects in the experiments. The RAGE agonists Advanced Glycation Endproduct-Bovine Serum Albumine (AGE-BSA) and S100 calcium binding protein B (S100B) were purchased from BioCat (Heidelberg, Germany) and Merck (Darmstadt, Germany). Forskolin and formyl-methionyl-leucyl-proline (fMLF) were obtained from Sigma-Aldrich, Munich, Germany.

### APP/PS1 transgenic mouse model

The APP/PS1 transgenic mouse model used in this study (APPswe/PS1dE9-Line 85) co-expresses a chimeric mouse/human amyloid precursor protein (APP) 695 harboring the Swedish K670M/N671L mutations (Mo/HuAPPswe) and human presenilin 1 (PS1) with the exon-9 deletion mutation (PS1dE9) under control of the mouse prion protein promoter [23]. The mouse line was obtained from Jackson Laboratory (B6.Cg-Tg(APPswe,PSEN1dE9)85Dbo/J; Stock-Number: 005864; Promoter: Prnp, prion protein; created by David Borchelt 2006, University of California, referring to Jackson Laboratory). Wildtype littermates were used as controls. Mice were used at 12 months of age. Mice were fed standard lab chow and water *ad libitum* and kept under a 12 h light/dark cycle.

### Cloning of cDNA and plasmids

The pcDNA3.1-hFPRL1 plasmid containing a neomycin resistance gene was kindly provided by Dr. U. Rescher (Münster, Germany). The pcDNA3.1-hFPR1 containing a neomycin resistance gene was obtained from UMS cDNA Resource Center (Rolla, Missouri, USA). The pcDNA3.1-hRAGE and –hRAGEΔcyto plasmids, containing a neomycin resistance gene, were kindly provided by Prof. R. Donato (Perugia, Italy). RAGEΔcyto (ΔRAGE) is a RAGE mutant lacking the cytoplasmic domain [24]. The inserts were subcloned into a pcDNA3.1 expression vector containing a Zeocin™ resistance gene (Invitrogen, Karlsruhe, Germany).

### Cell culture

HEK293 cells (American Type Culture Collection, Rockville, MD, USA) were subcultivated in Dulbecco’s modified Eagle’s medium (DMEM; PAA Laboratories, Pasching, Austria) supplemented with 10% fetal calf serum (FCS) and 1% penicillin/streptomycin (Carl Roth, Karlsruhe, Germany). The transfection and selection of HEK293 cells expressing hFPRL1 was described previously [11]. Using Lipofectamine 2000 (Invitrogen, Karlsruhe, Germany) according to the manufacturer’s protocol, HEK293 cells (American Type Culture Collection, Rockville, MD, USA) were first transfected with either pcDNA3.1-hFPR1 or –hFPRL1 plasmid. Stable transfectants were selected in the presence of 500 μg/ml G418 (Carl Roth, Karlsruhe, Germany). To generate hFPR1/hFPRL1 cell lines co-expressing hRAGE or hRAGEΔcyto, cells were subjected to a second round of transfection with pcDNA3.1-hRAGE or -hRAGEΔcyto and selected in the presence of 100 μg/ml Zeocin™ (Invitrogen, Karlsruhe, Germany).

Isolated cerebral cortices and rostral mesencephali from wistar rats (P2) were stripped of the meninges, minced and dissociated enzymatically with trypsin from bovine pancreas (Sigma-Aldrich, Taufkirchen, Germany) in phosphate-buffered saline and 50 μg/ml DNase I (Roche Molecular Biochemicals, Mannheim, Germany) for 30 min at 37°C and crushed mechanically with Pasteur pipettes. Astrocytes were prepared according to the protocol of McCarthy and DeVellis [25], which allows the preparation of nearly pure cultures of astrocytes (> 97%) and cultivated in Dulbecco’s modified Eagle’s medium (DMEM; PAA Laboratories, Pasching, Austria) supplemented with 10% FCS. Suspended microglial cells were plated in 75 cm^2^ cell culture flasks (Sarstedt, Nümbrecht, Germany) in microglial cell growth medium and harvested as described previously [13]. The microglial cell growth medium (DMEM) containing 10% FCS (heat inactivating from 44-53°C) and antibiotics (penicillin and streptomycin). After about ten days, the cells begin to move away from the cell layer and swim in the supernatant. The cells are collected and then seeded in normal medium (DMEM, 10% FCS heat inactivated at 56°C, penicillin and streptomycin). Prior to replating microglial cells for different assays, cell number and viability were estimated by trypan blue exclusion. This procedure increased the purity of the microglial preparation to > 98% with only very few remaining astrocytes.

### RNA isolation and real time RT-PCR

Total RNA was isolated using the peqGold Trifast reagent (Peqlab, Erlangen, Germany) according to the manufacturer’s instructions. RNA samples were reverse-transcribed by moloney murine leukemia virus (MMLV) reverse transcriptase (Fermentas, Burlington, Canada) and random hexamer primers (Invitrogen, Darmstadt, Germany). The cDNA products were used immediately for SYBR green (Applied Biosystems, Darmstadt, Germany) real-time RT-PCR for mus(m)FPR1, mFPR2 and RAGE. Gene expression was monitored using the StepOne Plus apparatus (Applied Biosystems, Darmstadt, Germany) according to manufacturer’s protocol [26]. Relative quantification was performed using the ΔCt method which results in ratios between target genes and a housekeeping reference gene (18 s). cDNA was amplified using gene-specific primers described in Table 1. The specificity of the amplification reaction was determined by a melting curve analysis. We performed relative quantification of the signals normalising to the Geomean of the gene signal from m18s, ribosomal protein L13a (RPL13a) and TATA box binding protein (TBP; all primers Eurofins MWG Operon, Ebersberg, Germany, for primer sequences please see Table 1) for SYBR Green real time RT-PCR.

**Table 1:** Primer sequences for real-time RT-PCR gene analysis

**primer**		**sequence**	**annealing Tm [°C]**
mFPR1	for	5’-CACAATCCAAGTCCGTGAACG-3’	57
	rev	5’-CAGCTGTTGAAGAAAGCCAAGG-3’	
mFPR2	for	5’-CTGAATGGATCAGAAGTGGTGG-3’	56
	rev	5’-CCCAAATCACTAGTCCATTGCC-3’	
mRAGE	for	5’-TGACCGCAGTGTAAAGAGTCCC-3’	59
	rev	5’-CCCTTAGCTGGCACTTAGATGG-3’	
m18s	for	5’-GAATAATGGAATAGGACCGCGG-3’	57
	rev	5’-AAGAATTTCACCTCTAGCGGCG-3’	
mTBP	for	5’-AGAACAATCCAGACTAGCAGCA-3’	58
	rev	5’-GGGAACTTCACATCACAGCTC-3’	
mRPL13a	for	5’-GAATAATGGAATAGGACCGCGG-3’	60
	rev	5’-GGCTCGGAATTGGTAGGGG-3’	

### Western blotting

For Western blot analysis of MAP kinase phosphorylation, rat glial or HEK293 cells were seeded in DMEM containing 10% FCS. Cells were harvested in a lysis buffer (50 mM Tris pH 7.5, 100 mM NaCl, 5 mM EDTA, 1% Triton, 2 mM sodium orthovanadate, 2.5 mM sodium pyrophosphate, 1 mM glycerol 2-phosphate, 1 mM phenylmethylsulfonylfluoride). Proteins (5 μg for pERK and ERK2) were resolved in SDS sample buffer, and a Western blotting procedure was performed as previously described in detail [12]. Membranes were incubated with polyclonal primary antibodies against pERK1/2 (1:500; sc-7383; Santa Cruz Biotechnology, Santa Cruz, CA, USA) overnight at 4°C and subsequent detection was performed with peroxidase-labeled secondary antibodies (Sigma-Aldrich, Munich, Germany). Antibody binding was detected by enhanced chemiluminescence (Amersham Pharmacia Biotech, Essex, UK). The membranes were then stripped and re-probed with anti-ERK2 (1:500; sc-1647; Santa Cruz Biotechnology, Santa Cruz, CA, USA) antibody as a loading control. The Western blot bands were densitometrically evaluated with the program Quantity One (Bio-Rad, Munich, Germany), the pERK-bands were adjusted with their respective ERK-bands and subsequently, the values were referred to control (=100%).

### Co-immunoprecipitation

Co-immunoprecipitation was performed as previously described in detail in Brandenburg et al. 2010 [10]. Cells (1.5 x 10^6^/plates for astrocytes and transfected HEK293 cells, 10 x 10^6^/plates for microglia) were plated onto 100 mm dishes and grown to 80% confluence. Cells were washed twice with phosphate-buffered saline and harvested into ice-cold lysis buffer (10 mM Tris–HCl, pH 7.6, 5 mM EDTA, 3 mM EGTA, 250 mM sucrose, 10 μM iodoacetamide, and a mixture of proteinase inhibitors: 0.2 mM phenylmethylsulfonyl fluoride, 10 μg/ml leupeptin, 1 μg/ml pepstatin A, 1 μg/ml aprotinin, and 10 μg/ml bacitracin). Subsequently, the cell suspensions were incubated for 30 min on ice and homogenised. The homogenates were then centrifuged at 500 g for 5 min at 4°C to remove not disrupted cells and nuclei. Membranes were pelleted at 20.000 g for 30 min at 4°C, and pellets were lysed in detergent buffer (20 mM HEPES, pH 7.4, 150 mM NaCl, 5 mM EDTA, 3 mM EGTA, 4 mg/ml β-dodecylmaltoside, and the proteinase inhibitors listed above) for 1 h on ice. Lysates were centrifuged at 20.000 g for 30 min at 4°C, and the protein content of the resulting supernatant was determined using a BCA protein assay (Pierce, Rockford, IL, USA). Receptor proteins were immunoprecipitated with 50 μl protein G agarose beads preloaded with 5 μg anti-FPR1 or anti-FPRL1 antibodies (for rat glial cells from Santa Cruz; for HEK cells transfected with hFPR1 or hFPRL1, from MBL, Woburn, MA, USA or Abcam (FPRL1, ab13177)) overnight at 4°C. Beads were washed five times with detergent buffer and eluted into 200 μl of SDS-sample buffer (62.5 mM Tris–HCl, pH 6.8, 2% SDS, 20% glycerol, 100 mM DL-dithiotreitol, and 0.005% bromphenol blue) at 60°C for 20 min. After SDS–polyacrylamide gel electrophoresis and electroblotting, membranes were incubated with rabbit anti-FPR1, FPRL1, RAGE (Abcam, Cambridge, UK; ab3611) antibodies overnight at 4°C. Immunoreactive bands were visualized using the enhanced chemiluminescence detection system mentioned above.

### Determination of receptor activity by measuring cyclic AMP accumulation

5 x 10^4^ astrocytes/well, or 7.5 x 10^4^ microglia/well or 1.5 x 10^4^ transfected HEK cells were seeded in a 96 well culture plate with DMEM containing 10% FCS and incubated for 48 h. The medium was removed and replaced with 100 μl of serum-free Opti-MEM medium containing 10 μM forskolin (for astrocytes, Sigma) or 25 μM forskolin (for microglial or HEK cells) plus agonist. Different forskolin concentrations were used because there is a difference in cell sensitivities to forskolin-stimulated adenylate cyclase activity. For the formyl peptide receptors antagonist WRW4, glial cells were pre-incubated in Opti-MEM medium containing 10 μM WRW4 for 30 min. The cells were incubated at 37°C for 15 min, and the reaction was terminated by the removal of the culture medium and addition of 70 μl lysis buffer (for HEK cells) or 100 μl lysis buffer (for glial cells) followed by 10 min incubation at room temperature. cAMP content was determined using a commercial available colorimetric kit (Millipore, Schwalbach, Germany).

### Fluorescence microscopy

Formalin-fixed and paraffin-embedded 5 μm whole coronary brain sections were examined. For immunofluorescence staining, sections were deparaffinized, pretreated for 3 x 7 min with microwaving in citric acid buffer, permeabilized with 0.1% Triton X in PBS for 10 min at room temperature and after blocking with 1.5% bovine serum albumine (BSA; Sigma-Aldrich, Taufkirchen, Germany) in TRIS incubated with either polyclonal rabbit anti-mFPR1 (1:100; ab101701; Abcam, Cambridge, UK), anti-mFPR2 (1:100; sc-18191; Santa Cruz Biotechnology), anti-RAGE (1:100; ab3611; Abcam, Cambridge, UK) and monoclonal mouse anti-GFAP (1:250; ab10062; Abcam, Cambridge, UK) or polyclonal goat anti-Iba1 (1:100; ab5076; Abcam, Cambridge, UK) overnight at 4°C. Finally, the slices were incubated with donkey anti-rabbit AlexaFluor 488 (Molecular Probes, Darmstadt, Germany) and goat anti mouse Cy3 (Sigma-Aldrich, Taufkirchen, Germany) or rabbit anti-goat AlexaFluor 555 (all 1:250; Molecular Probes, Darmstadt, Germany) for 1 h at room temperature.

### Fluorescence staining of primary and cell cultures

Glial or HEK293 cells were grown on glass coverslips. Coverslips were previously coated with poly-L-lysine according the instruction (Sigma-Aldrich, Munich, Germany). Transfected HEK293 cells were exposed to RAGE agonist S100B (5 μg/ml = 2.4 μM) for 2 h at 37°C. After fixation with 4% paraformaldehyde and 0.2% picric acid in a phosphate buffer at pH 6.9 [11] for 30 min and permeabilisation with 0.1% TritonX in PBS, cells were blocked in 0.1 M Tris–HCl pH 7.5 containing 1.5% BSA for 10 min. Coverslips were incubated at 4°C overnight with primary antibodies for FPR, FPRL1 or RAGE (for rat glial cells: FPR/FPRL1 from Santa Cruz; RAGE from AbD Serotec, Düsseldorf, Germany (goat) or Abcam (rabbit); for transfected HEK cells: hFPR1 from MBL (rabbit) or Santa Cruz (goat); hFPRL1 from Abcam (rabbit) or Everest Biotech, Oxfordshire, UK (goat); RAGE from AbD Serotec and S100B from Abcam (ab868, rabbit); Aβ1–42 from Santa Cruz (sc-58495, mouse) and diluted in TRIS containing 1.5% BSA. Finally, the coverslips were incubated with donkey anti-rabbit or anti-goat AlexaFluor 488 (Molecular Probes, Darmstadt, Germany) and goat anti rabbit Cy3 (Millipore) or rabbit anti-goat AlexaFluor 555 (all 1:250; Molecular Probes, Darmstadt, Germany) for 1 h at room temperature. Nuclear staining was performed with bisbenzimide (Sigma-Aldrich, Taufkirchen, Germany). Cells were digitally photographed using a LSM7 DUO laser confocal microscope (Zeiss, Göttingen, Germany).

### Determination of co-localisation

The Pearson coefficient is a measure of the strength of linear relationship between two signals. Instead, Spearman coefficient is a measure of how well any monomeric function between the variables can describe the relationship. The Pearson-Spearman correlation (PSC) co-localisation plugin for ImageJ was used to calculate co-localisation between target receptors RAGE, FPR1 or FPRL1 and S100B or Aβ1-42 [27,28]. A subselection as a region of interest (ROI) was set up around the plasma membrane using the Selection Brush with a width of 25 pixels. The value for the background intensity noise threshold was set up to 40 to calculate the coefficients.

### Statistical analysis

All *in vitro* experiments were performed at least in triplicate and the values are expressed as mean ± SEM. For statistical comparison, ANOVA test was used followed by Bonferroni’s correction. A value of p < 0.05 was considered statistically significant. The GraphPad Prism 5.0 software was used for statistical calculation (Graph Pad Software, San Diego, CA, USA).

## Results

### Increased formyl peptide receptors and RAGE expression in glial cells in an APP/PS1 transgenic mouse model

**Figure 1:**
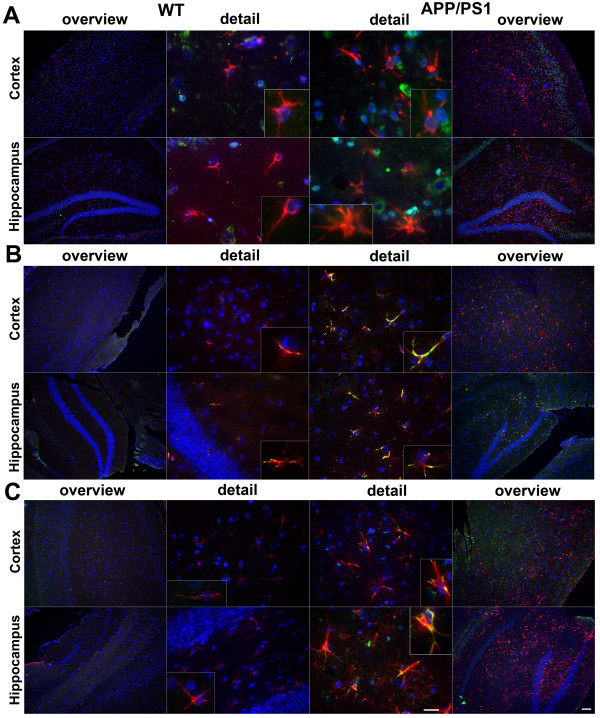
**Increased formyl peptide receptors and RAGE expression in astrocytes in a APP/PS1 transgenic mouse model.** Coronal brain sections from twelve month old APPswe/PS1dE9 (APP/PS1) or wildtype (WT) mice were stained with anti-glial fibrillary acidic protein (GFAP) to identify astrocytes (red) and anti-mFPR1 (**A**), anti-mFPR2 (**B**) or anti-RAGE (**C**) (green) antibodies (nuclear counterstaining in blue). The figures show representative results for the cortex and hippocampus from one of three independent experiments (Scale bar = 200 μm for overview and 20 μm for detailed images).

**Figure 2:**
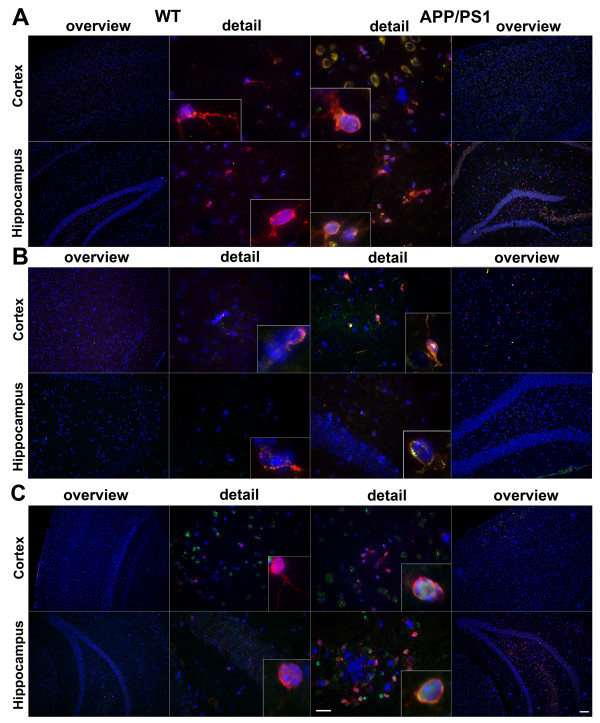
**Increased formyl peptide receptors and RAGE expression in microglial cells in a APP/PS1 transgenic mouse model.** Coronal brain sections from twelve month old APPswe/PS1dE9 (APP/PS1) or wildtype (WT) mice were stained with anti-ionized calcium binding adaptor molecule-1 (anti-Iba-1) to identify microglial cells (red) and anti-mFPR1 (**A**), anti-mFPR2 (**B**) or anti-RAGE (**C**) (green) antibodies (nuclear counterstaining in blue). The figures show representative results for the cortex and hippocampus from one of three independent experiments (Scale bar = 200 μm for overview and 20 μm for detailed images).

In a first set of experiments we investigated the expression and localisation of the mouse formyl peptide receptors mFPR1 and mFPR2 as well as RAGE in an APP/PS1 transgenic mouse model of AD. We used double fluorescence microscopy with receptor and glial cell (GFAP for activated astrocytes and Iba-1 for microglia/macrophages) specific antibodies to localise the receptor expression in mouse brain sections. The receptor expression was analysed in the cortex and hippocampus of twelve month old double transgenic mice co-expressing human PS1dE9 and mouse/human (mo/hu) chimeric APP695 (humanized Aβ domain) harboring the Swedish (K594M/N595L) mutation [23] and compared with wildtype littermates. As shown in figure 1 and 2, only few GFAP- and Iba-1-immunoreactive cells and very little mFPR1 (Figures 1A and 2A), mFPR2 (Figures 1B and 2B) as well as RAGE (Figures 1C and 2C) immunoreactivity were detected in the wildtype cortex and hippocampus. In contrast, the brain slices of contemporary APP/PS1 transgenic mice showed a strong increase of both GFAP and Iba-1 immunoreactivity in the cortex and hippocampus. For mFPR1, in the cortex the GFAP positive cells showed a slight and in the hippocampus a clear increase of mFPR1 immunofluorescence (Figure 1A). A significant mFPR1 expression in brain slices of cortex and hippocampus co-localised with Iba1-positive cells was detected (Figure 2A). For mFPR2, a strong increase of immunoreactivity was evident in GFAP- as well as Iba-1-positive cells in the cortex and hippocampus of APP/PS1 transgenic mice (Figures 1B and 2B). For RAGE, the immunoreactivity was strongest in the hippocampus in GFAP- and Iba1-positive cells, but also in the cortex, an increase was detected (Figures 1C and 2C). Please not that we did not have observe a clear increase of receptor expession in the near of the plaques.

**Figure 3:**
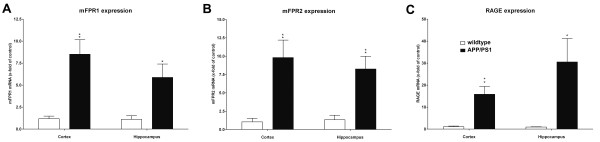
**Increased receptor expression in the cortex and hippocampus of twelve month old APP/PS1 transgenic mice.** The cortex and hippocampus from twelve month old APP/PS1 or wildtype mice were isolated and mRNA expression of mFPR1 (**A**), mFPR2 (**B**) or RAGE (**C**) were determined using realtime RT-PCR. Data were assessed from six independent experiments in duplicate. An asterisk indicates a significant difference (* = p < 0.05; ** = p < 0.01) compared to wildtype mice as determined by ANOVA followed by Bonferroni test.

To confirm the immunofluorescence studies, the mRNA expression of mFPR1, mFPR2 and RAGE in the two brain regions cortex and hippocampus was quantified using real- time RT-PCR in APP/PS1 transgenic mice compared to wildtype littermates. As shown in Figure 3A, the mFPR1 mRNA expression was significantly increased in the hippocampus and cortex of the APP/PS1 transgenic mice (cortex: 8.5 ± 1.6 fold increase; hippocampus: 5.9 ± 1,5 fold increase, p < 0.01 and p < 0.05; Figure 3A). For mFPR2, the expression was significantly increased in the cortex as well as the hippocampus (cortex: 9.8 ± 2.3 fold increase; hippocampus: 8.3 ± 1.7 fold increase, both p < 0.01; Figure 3B). For RAGE, the maximum induction of expression was detected in the hippocampus, whereas in the cortex also a significant increase was determined (cortex: 15.8 ± 3.5 fold increase; hippocampus: 30.5 ± 10.6 fold increase, p < 0.01 and p < 0.05; Figure 3C).

### Inhibition of RAGE-induced signal transduction by formyl peptide receptor antagonist WRW4 in glial cells

**Figure 4:**
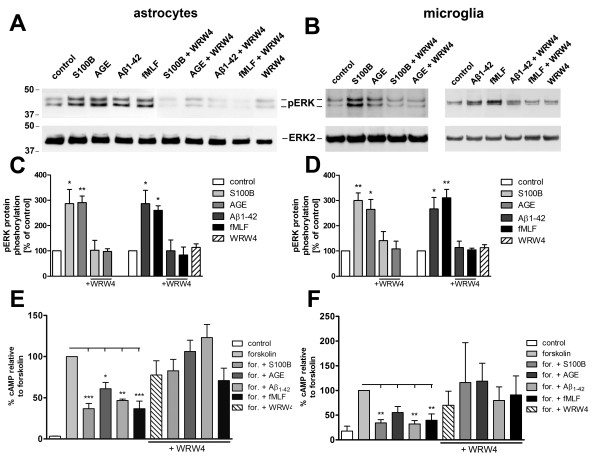
**Inhibition of RAGE-mediated G-protein receptor activity by the formyl peptide receptor antagonist WRW4 in glial cells.** For analysis of ERK1/2 phosphorylation, astrocytes (**A**) and microglia (**B**) were each treated with 1 μM Aβ1–42, 1 μM fMLF, 2.4 μM S100B or 0.75 μM AGE-BSA (AGE) with or without 10 μM WRW4 and with WRW4 alone for 5 min at 37°C. Cells were lysed, equal amounts of protein (5 μg) were dissolved in SDS sample buffer, and the levels of total ERK2 and phosphorylated phosphorylated ERK1/2 were determined via immunoblotting. The positions of phospho-ERK1/2 (pERK1/2) and total ERK2 (ERK2) along with those of the molecular mass markers (in kDa) are indicated on the left side. The values representing mean ± standard error of the mean (SEM) of phosphorylation levels derived from densitometric quantification of three independent experiments are indicated in (**C**) and (**D**). An asterisk indicates a significant difference (* - p < 0.05) compared to control as determined by one-way ANOVA and Bonferroni post-hoc test. In order to analyse the inhibition of forskolin-stimulated adenylate cyclase activity, astrocytes (**E**) and microglia (**F**) were subjected to either 10 μM (**E**) or 25 μM (**F**) forskolin as well as well as to 1 μM Aβ1–42, 1 μM fMLF, 2.4 μM S100B or 0.75 μM AGE-BSA (AGE) with or without 10 lM WRW4 and to WRW4 alone for 15 min at 37°C. cAMP levels were determined as described above (see Methods). The values given represent mean ± SEM from four independent experiments. Asterisks indicate significant differences (*, p < 0.05) between forskolin plus agonists and forskolin alone as determined by one-way ANOVA and Bonferroni post-hoc tests.

We were able to detect an increased receptor expression for formyl peptide receptors (mFPR1 and mFPR2) and RAGE in the APPswe/PS1dE9 mice. In the next step we investigated the glial cell activation by the different receptor ligands. Therefore, we incubated primary rat astrocytes and microglial cells with Aβ1–42 and fMLF as formyl peptide receptor agonists and S100B and AGE-BSA as RAGE agonists to determine ERK1/2 phosphorylation and the inhibition of forskolin-induced cAMP accumulation. The rat glial cells express FPR1 (homologous to mFPR1) and FPRL1 (homologous to mFPR2) [11]. As shown in Figure 4A to D, treatment with Aβ1–42 and fMLF as well as S100B and AGE-BSA resulted in an intense phosphorylation of ERK1/2 in glial cells. The formyl peptide receptor antagonist WRW4 inhibited the Aβ1–42- and fMLF-induced ERK1/2 phosphorylation in astrocytes as well as microglia cells. This confirmed our previous results [11,13]. In addition, the present work showed that the S100B- and AGE-BSA-induced ERK1/2 phosphorylation is also inhibited by WRW4, which has no effect on ERK1/2 phosphorylation by itself. In addition to the ERK1/2 phosphorylation mentioned, the formyl peptide receptors capacity to inhibit cAMP formation was investigated. The formyl peptide receptors are coupled to inhibitory G-protein (G_i_). The activation resulted in a reduction of cAMP level. Forskolin was used as the activator of the adenylate cyclase [12]. To determine whether the different agonists inducing cAMP formation is linked via G_i_ receptor activity, the cAMP production in glial cells was induced by forskolin treatment, and the interference of Aβ1–42, fMLF, S100B and AGE-BSA was analysed. As shown in Figure 4E and F, the treatment with forskolin resulted in a strong increase of intracellular cAMP for astrocytes (up to 56 ± 18 pmol/ml) and microglia (up to 38 ± 15 pmol/ml) as compared to untreated cells. The change in the amount of cAMP was calculated as a percentage relative to forskolin. Application of both Aβ1–42 and fMLF counteracted the forskolin-induced cAMP formation in glial cells and the decrease of cAMP level was strongly inhibited by the formyl peptide receptors antagonist WRW4 [22]. This confirmed our previous results [11,13]. In addition, also S100B as well as AGE-BSA reduced forskolin-induced cAMP formation in astrocytes and microglial cells (Figure 4E and F). Moreover, the S100B- and AGE-BSA-induced decrease of cAMP level was also blocked by the formyl peptide receptors antagonist WRW4. These results suggest that FPR1/FPRL1 is involved in RAGE signalling. WRW4 alone did not alter the forskolin-stimulated adenylate cyclase activity in glial cells.

### Detection of the co-localization of RAGE and FPR1 as well as FPRL1 in rat glial and transfected HEK293 cells by immunofluorescence

**Figure 5:**
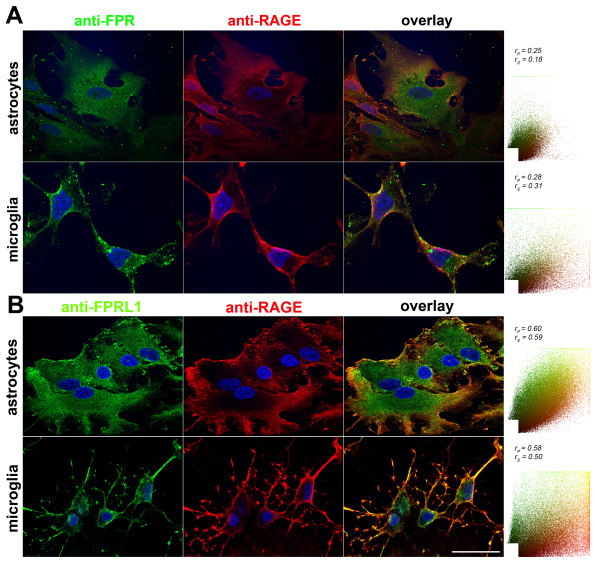
**FPR1 and FPRL1 are co-localised with RAGE in primary rat glial cells.** Astrocytes and microglial cells were fixed and labelled with anti-FPR1 (**A**) or anti-FPRL1 (**B**) and anti-RAGE antibodies. Localisation of FPR1, FPRL1 and RAGE was examined by double fluorescence microscopy. Bisbenzimide was used for nuclear counter-staining (blue). The figures show representative results from one of three independent experiments, each performed in duplicate. Scale bar: 20 μm. The right columns showed resulting scatter plot of intensities across the two channels. The Pearson correlation coefficient r_p_ and Spearman correlation coefficient r_s_ are indicated on the scatter plots. For FPR1 (green channel) and RAGE (red channel) (**A**), low co-localisation and for FPRL1 (green channel) and RAGE (red channel) (**B**) good co-localisation.

As described above, S100B- and AGE-BSA-induced ERK1/2 phosphorylation and the reduction of cAMP levels were inhibited by the formyl peptide receptors antagonist WRW4. One can assume that the effects of RAGE are partly mediated by FPR1/FPRL1. To further substantiate our findings, we examined the distribution of FPR1, FPRL1 and RAGE in rat glial cells using double fluorescence microscopy with receptor-specific antibodies. For FPR1 and RAGE, both receptors are co-localised mainly in the plasma membrane in astrocytes as well as microglial cells (Figure 5A). Also FPRL1 showed overlapping staining with RAGE in the plasma membrane of glial cells (Figure 5B). Next, we quantified the co-localisation of fluorescent markers (see methods). The right columns showed the results of a scatter plot of intensities across the two channels (FPR1/FPRL1, green and RAGE, red). Coefficients of correlations are presented over the scatter plots. The range is from −1, a strong negative correlation, to +1, a strong positive correlation. The closer to zero a coefficient is, the weaker the correlation and hence the less evidence there is for co-localisation. The Pearson correlation coefficient r_p_ and Spearman correlation coefficient r_s_ are indicated on the scatter plots. Between FPR1 and RAGE in astrocytes as well as microglial cells, the coefficients illustrate a low co-localisation. For FPRL1 and RAGE, the coefficients confirm a good co-localization.

**Figure 6:**
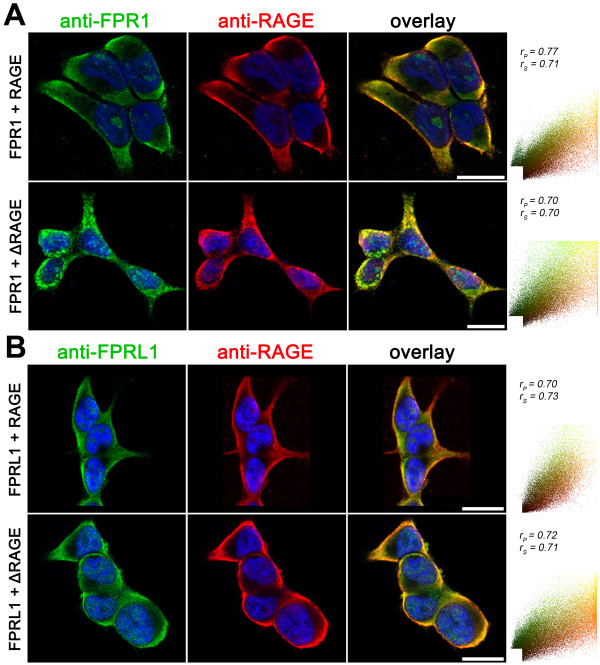
**FPR1 and FPRL1 are co-localised with RAGE in transfected HEK293 cells.** FPR1 (**A**) or FPRL1 (**B**) and RAGE or ΔRAGE transfected HEK293 cells were fixed and labelled with anti-FPR1 (**A**) or anti-FPRL1 (**B**) and anti-RAGE antibodies. Localisation of FPR1, FPRL1 and RAGE/ΔRAGE was examined by double fluorescence microscopy. Bisbenzimide was used for nuclear counter-staining (blue). The figures show representative results from one of three independent experiments, each performed in duplicate. Scale bar: 20 μm. The right columns show the resulting scatter plot of intensities across the two channels. The Pearson correlation coefficient r_p_ and Spearman correlation coefficient r_s_ are indicated on the scatter plots. For FPR1/FPRL1 (green channel) and RAGE/ΔRAGE (red channel), please note a good co-localisation.

Furthermore, we analysed the FPR1/FPRL1 and RAGE interaction in transfected HEK293 cells by fluorescence microscopy. Therefore, we stably expressed human FPR1 or FPRL1 and RAGE or a RAGE mutant lacking the cytoplasmic and transducing domain (ΔRAGE) in HEK293 cells. As shown in Figure 6A and B, the FPR1 as well as FPRL1 and RAGE are strongly co-localised in plasma membrane. The co-localisation was also quantified and the resulting scatter plot is showed in the right column. The coefficients illustrate a good co-localisation between FPR1 as well as FPRL1 and RAGE as well as ΔRAGE. We were not able to detect a difference between RAGE and ΔRAGE for co-localization.

### RAGE interacts with FPR1 and FPRL1 in rat glial and transfected HEK293 cells

**Figure 7:**
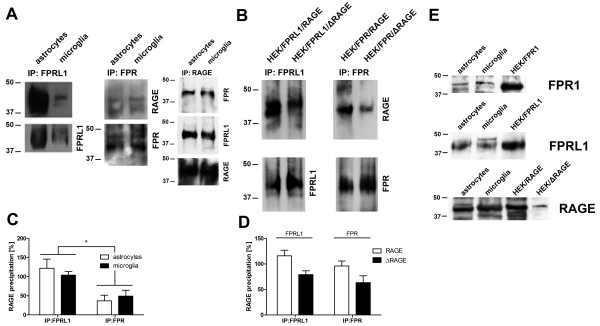
**RAGE interacts with FPR1 and FPRL1 in glial and transfected HEK293 cells.** (**A**) Membrane proteins from astrocytes or microglia were extracted using anti-ratFPRL1 or ratFPR1 antibodies. (**B**) Membrane proteins from FPR1 or FPRL1 and RAGE or ΔRAGE transfected HEK293 cells were extracted and anti-humanFPRL1 or humanFPR1 antibodies were used to precipitate. The resulting immunoprecipitates were electrophoretically separated, transferred to nitrocellulose and detected with anti-FPR1, anti-FPRL1 and anti-RAGE antibodies. FPR1 and FPRL1 are co-immunoprecipitate with RAGE in glial as well as transfected HEK293 cells. The positions of molecular mass markers are indicated on the left (in kDa). The values representing mean ± SD of protein and phosphorylation levels derive from densitometric quantification of three independent experiments for co-immunoprecipitated RAGE (**C** for glial cells and **D** for transfected HEK293 cells) normalised to FPR1 or FPRL1. An asterisk indicates a significant difference (*p < 0.05) compared to controls on the basis of one-way ANOVA and Bonferroni post-hoc tests. (**E**) Western Blotting for different receptors of whole cell lysate from astrocytes, microglial and transfected HEK293 cells (each 10 μg protein). The positions of molecular mass markers are indicated on the left (in kDa) and the target on the right.

To confirm our immunofluorescence results concerning FPR1/FPRL1 and RAGE interaction, co-immunoprecipitation studies were conducted using formyl peptide receptor FPR1 or FPRL1 and RAGE antibodies. FPR1 or FPRL1 receptors were precipitated from lysates of rat glial cells using anti-FPR1 or FPRL1 antibodies. The precipitates were immunoblotted with antibodies directed against FPR1, FPRL1 or RAGE. As shown in Figure 7A RAGE was detected in immunoprecipitates from astrocytes and microglial cells, suggesting that FPR1 or FPRL1 is physically associated with RAGE *in vitro* and/or under the experimental conditions described. The band densities of the western blots were evaluated by densitometric quantification. As shown in Figure 7C, we did not detect a difference between astrocytes and microglial cells for RAGE in FPRL1 as well as FPR1 precipitates, but the amount of co-immunoprecipitated RAGE was higher in FPRL1 precipitates compared to FPR1 precipitates. To confirm the immunoprecipitation, we precipitate RAGE from lysate of astrocytes or microglia. As shown in Figure 7A, FPR1 and FPRL1 were detected in the immunprecipitates.

Furthermore, we analysed the FPR1/FPRL1 and RAGE interaction in transfected HEK293 cells by co-immunoprecipitation. Co-immunoprecipitation studies were conducted with anti-human FPR1 or FPRL1 antibodies using lysates of FPR1/RAGE as well as FPR1/ΔRAGE or FPRL1-RAGE as well as FPRL1/ΔRAGE expressing HEK293 cells. The precipitates were immunoblotted with antibodies directed against FPR1, FPRL1 or RAGE. As shown in Figure 7B, RAGE was detected in immunoprecipitates from co-transfected HEK293 cells, which confirms that FPR1 and FPRL1 are physically associated with RAGE in vitro. The mutant receptor ΔRAGE was also detected in the FPR1 as well as FPRL1 precipitate. The densitometric quantification of the band densities showed a significant decrease of the amount of co-immunoprecipitated RAGE in the FPR1/ΔRAGE as well as FPRL1/ΔRAGE expressing HEK293 cells (Figure 7D).

Next, the protein receptor expression in the cell lysates of glial and transfected HEK293 cells were determined. As shown in Figure 7E, FPR1, FPRL1 and RAGE protein expression were detected in astrocytes, microglia and the corresponding transfected HEK293 cells.

### FPR1-, FPRL1- and RAGE-mediated ERK1/2 phosphorylation and change of cAMP levels in transfected HEK293 cells

**Figure 8:**
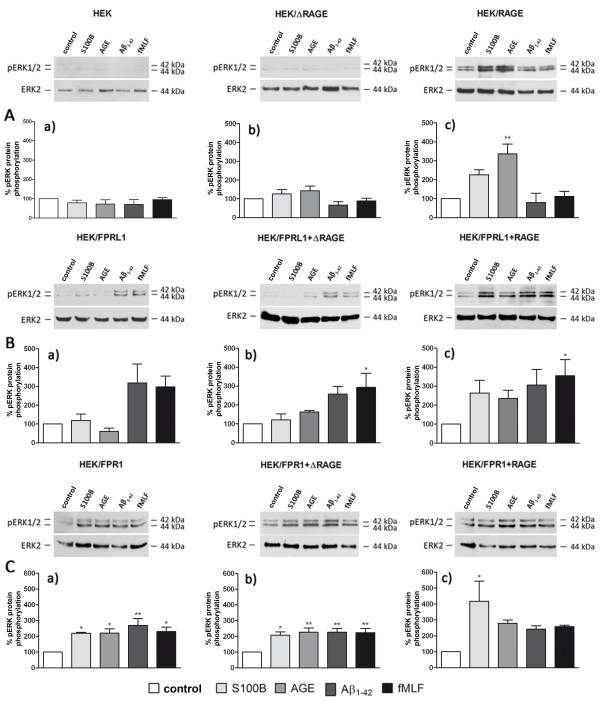
**FPR1-, FPRL1- and RAGE-mediated ERK1/2 phosphorylation in transfected HEK293 cells.** For analysis of ERK1/2 phosphorylation, (**A**) untransfected (**a**) or ΔRAGE (**b**), RAGE (**c**); (**B**) FPRL1 (**a**) and ΔRAGE (**b**) or RAGE (**c**); (**C**) FPR1 (**a**) and ΔRAGE (**b**) or RAGE (**c**) expressing HEK293 cells were treated with 1 μM Aβ1–42, 1 μM fMLF, 2.4 μM S100B or 0.75 μM AGE-BSA (AGE) for 5 min at 37°C. Cells were lysed, equal amounts of protein (5 μg) were dissolved by SDS sample buffer, and levels of total ERK2 and phosphorylated ERK1/2 were determined by immunoblotting. The positions of molecular mass markers are indicated on the right (in kDa). The mean ± SD of the three independent experiments were evaluated by densitometric quantification normalised to ERK2 expression. Asterisks indicate a significant difference (*, p < 0.05; **, p < 0,001) compared to control (one-way ANOVA followed by the Bonferroni test).

In an additional set of experiments, we investigated the effect of FPR1, FPRL1 and RAGE on Aβ1–42-, fMLF-, S100B and AGE-BSA-induced signal transduction in transfected HEK293 cells. For this purpose, we generated FPR1-, FPRL1-, RAGE or ΔRAGE-expressing, and FPR1 or FPRL1 and RAGE or ΔRAGE-co-expressing HEK293 cells and analysed ERK1/2 phosphorylation after Aβ1–42-, fMLF-, S100B and AGE-BSA-treatment. The results of the Western blots were quantified by densitometric quantification. In un-transfected and ΔRAGE-expressing HEK293 cells, no stimulant resulted in an increase of the ERK1/2 phosphorylation (Figure 8A(a and b)). For RAGE-expressing HEK293 cells, S100B as well as AGE-BSA induced an increase of ERK1/2 phosphorylation in RAGE-expressing cells, whereas Aβ1-42 and fMLF showed no effect (Figure 8A(c)). In FPRL1-transfected and FPRL1-ΔRAGE-co-expressing HEK293 cells, only Aβ1–42 and fMLF induced an increase of ERK1/2 phosphorylation (Figure 8B(a and b)), whereas in FPRL1-RAGE-co-expressing HEK293 cells, Aβ1–42, fMLF, S100B and AGE-BSA significantly increased ERK1/2 phosphorylation (Figure 8B(c)). Interestingly, in FPR1-expressing and FPR1-ΔRAGE-co-expressing HEK293 cells, Aβ1–42- and fMLF- as well as S100B and AGE-BSA induced an increase of ERK1/2 phosphorylation (Figure 8C(a and b)). Furthermore, also in FPR1-RAGE-co-expressing HEK293 cells, Aβ1–42, fMLF, S100B and AGE-BSA increased a significantly ERK1/2 phosphorylation (Figure 8C(c)).

**Figure 9:**
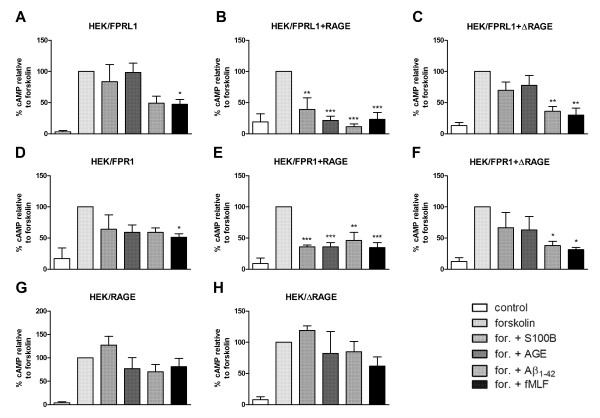
**FPR1-, FPRL1- and RAGE-mediated change of cAMP levels in transfected HEK293 cells.** For analysis of inhibition of forskolin-stimulated adenylate cyclase activity, FPRL1 (**A**) and RAGE (**B**) or ΔRAGE (**C**); FPR1 (**D**) and RAGE (**E**) or ΔRAGE (**F**); and RAGE (**G**) or ΔRAGE (**H**) expressing HEK293 cells were subjected to 25 μM forskolin as well as 1 μM Aβ1–42, 1 μM fMLF, 2.4 μM S100B or 0.75 μM AGE-BSA (AGE) for 15 min at 37°C. cAMP levels were determined as described above (see Methods). The values represent mean ± SEM from four independent experiments. Asterisks indicate a significant difference (*, p < 0.05; **, p < 0,01) between forskolin plus agonists and forskolin alone, as determined by one-way ANOVA followed by the Bonferroni test.

The above results are reflected in a change in forskolin-induced adenylate cyclase activity. In hFPRL1-expressing and FPRL1-ΔRAGE-co-expressing cells only the stimulation with Aβ1–42 and fMLF were able to reduce the cAMP level significantly (Figure 9A and C), whereas in FPRL1-RAGE-co-expressing HEK293 cells also S100B and AGE-BSA showed an effect on cAMP level (Figure 9B). Interestingly, in FPR1-expressing HEK293 cells, Aβ1–42- and fMLF- as well as AGE-BSA induced attenuated forskolin-induced cAMP accumulation (Figure 9D). S100B induced a slight but not significant reduction of forskolin-induced adenylate cyclase activation. In FPR1-RAGE-co-expressing HEK293 cells Aβ1–42, fMLF, S100B and AGE-BSA significantly reduced forskolin-induced cAMP level. In contrast, co-expression with ΔRAGE cancels the effect of S100B and AGE-BSA compared to Aβ1–42- as well as fMLF-mediated inhibition of forskolin-induced cAMP accumulation (Figure 9E and F). In HEK293 cells, only transfected with RAGE or ΔRAGE, neither of the agonists induced a change of forskolin-induced adenylate cyclase activation (Figure 9G and H).

### Different receptors and S100B as well as Aβ1–42 co-localization in transfected HEK293 cells

**Figure 10:**
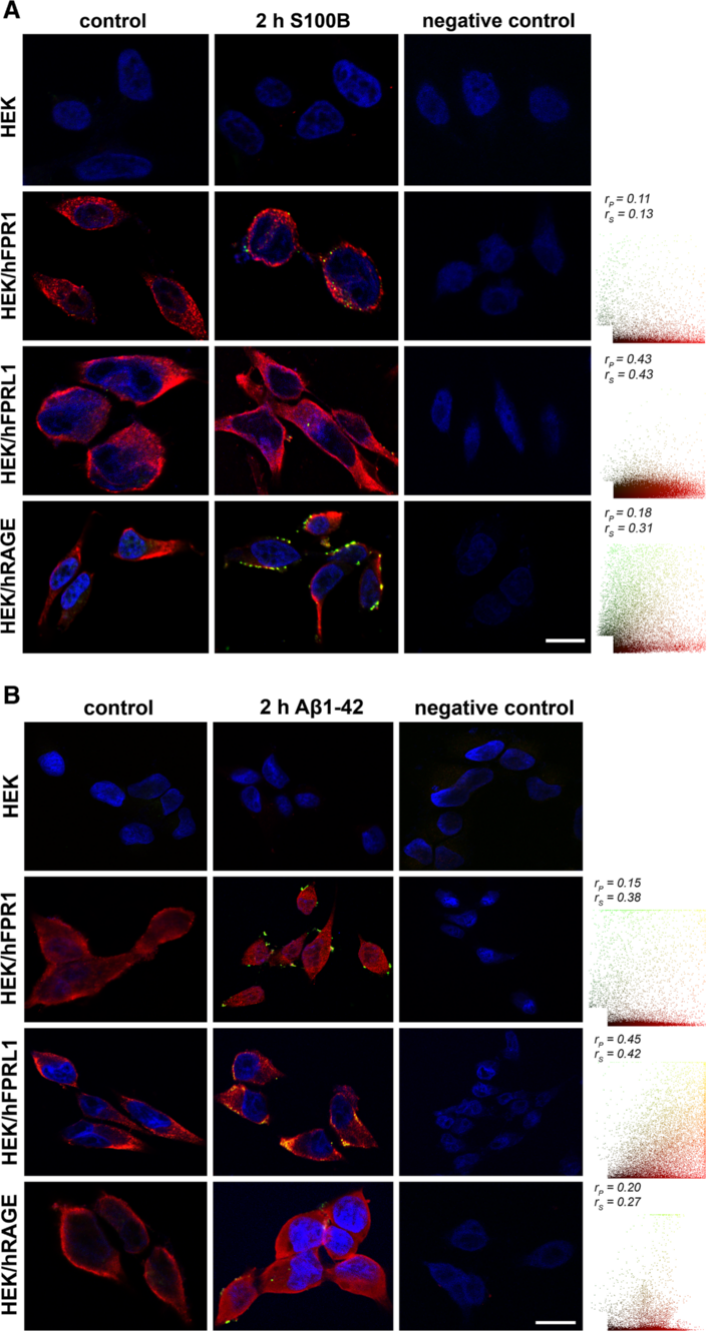
**Different receptors and S100B or Aβ1–42 co-localisation in transfected HEK293 cells.** Untransfected or FPR1-, FPRL1- or RAGE expressing HEK293 cells were incubated with 2.4 μM S100B or 1 μM Aβ1–42 for 2 h. Cells were fixed and labelled with anti-FPR1, -FPRL1, -RAGE and anti-S100B or anti- Aβ1–42 antibodies. Localisation of FPR1, FPRL1 or RAGE and S100B (**A**) or Aβ1–42 (**B**) was examined by double fluorescence microscopy. Bisbenzimide was used for nuclear counter-staining (blue). The figures show representative results from one of three independent experiments, each performed in duplicate. Scale bar: 20 μm. The right columns show the resulting scatter plot of intensities across the two channels. The Pearson correlation coefficient r_p_ and Spearman correlation coefficient r_s_ are indicated on the scatter plots. For FPR1 (red channel) and S100B or Aβ1–42 (green channel), poor or low co-localisation; for FPRL1 (red channel) and S100B or Aβ1–42 (green channel), low or good co-localisation and for RAGE (red channel) and S100B or Aβ1–42 (green channel), both low co-localisation.

After we were able to demonstrate an involvement of the formyl peptide receptors in S100B- as well as AGE-BSA-induced signal transduction, we investigated whether a direct interaction between S100B or Aβ1–42 and the receptors is detectable. Therefore, we analysed and quantified the co-localisation of different receptors with S100B as well as Aβ1-42 using fluorescence microscopy after treatment of different receptor-expressing HEK293 cells with S100B or Aβ1–42 for 2 h. As shown in Figure 10A, in untranfected HEK293 cells we did not determine a detectable amount of bound S100B. In FPR1 expressing HEK293 cells, a slight increase of S100B in the cells was detectable, whereas in FPRL1 expressing HEK293 some S100B particles were identified. The largest amount of S100B was detected in RAGE expression HEK293 cells. The co-localisation was also quantified and the resulting scatter plot is in the right column. The coefficients illustrate a low but existing co-localisation between FPRL1 as well as RAGE and S100B. Between FPR1 and S100B, the coefficients illustrate a poor co-localisation.

For the Aβ1–42 and the different receptors, the co-localisation was also quantified. The results in Figure 10B show a good co-localisation between Aβ1–42 and FPRL1, whereas the coefficients illustrate a low but existing co-localisation between FPR1 as well as RAGE and Aβ1–42.

## Discussion

Our study shows the involvement of formyl peptide receptors FPR1 and FPRL1 in Aβ1-42-induced signal transduction in glial and transfected HEK293 cells. This confirmed and extended our previous results for the FPRL1 [11,13]. Interestingly, our results also show an involvement of the high affinity receptor FPR1 in Aβ1-42-induced signal transduction in transfected HEK293 cells. A previous result from Le et al. [29] reported that Aβ1–42 is able to activate FPR1 in transfected HEK293 and a rat basophilic leukemia cell line, but that the receptor’s efficacy in mediating cell migration and activation is much lower than that of FPRL1. In our transfected HEK293 cells, we did not observe a clear difference between FPR1 and FPRL1 expressing cells in Aβ1-42-induced ERK1/2 phosphorylation or a change of cAMP accumulation (Figures 8 and 9). Our previous results with small inferring RNA against FPR1 in primary astrocytes did not result in an inhibition of Aβ1-42-induced ERK1/2 phosphorylation. However, the results could be explained by the low FPR1 expression in the astrocytes as our previous results had shown [11]. It should be noted that microglial cells show a higher endogen FPR1 expression. In addition, other receptors are discussed for Aβ1-42-induced glial cell activation. Other groups have reported that the scavenger receptor MARCO (macrophage receptor with collagenous structure), a cell surface glycoprotein, plays a role in the internalisation and Aβ1–42-mediated microglia activation [16]. Our previous results in astrocytes and transfected HEK293 cells did not show an involvement of MARCO in Aβ1-42-induced ERK1/2 phosphorylation and change of cAMP accumulation [11]. Recent works suggested that scavenger receptors mediate Aβ internalisation in microglial cells or activation of perivascular macrophages [30,31]. A further receptor, which is discussed in the context of the Aβ1-42-induced glial cell activation, is RAGE. Previous studies had shown that RAGE binds Aβ1–42 with high affinity in microglial cells and neurons [17]. It was suggested that RAGE-dependent signaling in microglial cells contributes to neuroinflammation and Aβ accumulation as well as impaired learning/memory in an APP/PS1 transgenic mouse model [32]. The crossing of these mice with an inactive RAGE mutant resulted in a decrease of Aβ levels and amyloid plaque load. It should be noted that other working groups were not able to determine an effect of RAGE in an APP/PS1 transgenic mouse model [33]. However, our present results show a strong increase of mice formyl peptide receptors mFPR1 and 2, the mice homologon to human FPR1 and FPRL1, as well as RAGE expression in the hippocampus and for mFPR2 also in the cortex of the used APP/PS1 transgenic mice (Figure 3). Nevertheless, it must be noted that nothing is known about the increase of receptor expression in the human AD brain. The increase was co-localised to astrocytes and microglia cells (Figures 1 and 2). Also for MARCO, we were able to detect a strong increase of expression co-localised to glial cells (data not shown). This extended previous results for RAGE and MARCO [34,35]. Altogether, the increasing receptor expression during the course of Aβ1-42 deposition in APP/PS1 transgenic mice could be a sign of enhanced inflammation including glial cell activation. The receptor activation of different signal transduction pathways including NADPH oxidase or NFκB is increased. This may be associated with an increased production of proinflammatory cytokines and reactive oxygen species [36-38]. However, a recent study showed that the mFPR2 acted as an anti-inflammatory receptor [39]. It could also be a sign of the increased uptake and clearance of Aβ. Our previous work showed the involvement of FPRL1 in glial cells mediated Aβ1-42 internalisation [13]. Possible, the increase of receptor expression represents a protective function against increased Aβ concentration in the brain. The receptor-mediated internalisation could be an interesting point to influence the plaque as well as AD development. In this context, previous results showed that the glial cells are able to internalise Aβ, although the uptake is dependant on Aβ forms and size [13,40-42]. Further studies must explore this topic.

Interestingly, in this study, we have demonstrated a physical interaction between FPR1 or FPRL1 and RAGE in glial and transfected HEK293 cells by co-immunoprecipitation and fluorescence microscopy for the first time (Figures 5,6,7). The densitometric quantification and quantitative statistical co-localisation show that the interaction was stronger between FPRL1 and RAGE compared to FPR1 and RAGE in glial cells. Differences between astrocytes, microglial cells and in transfected HEK293 cells were detectable. In FPR1 as well as FPRL1 and ΔRAGE, a RAGE mutant lacking the cytoplasmic and transducing domain, co-expressing HEK293 cells, showed that the interaction was significantly but not completely reduced (Figure 7). The intracellular domain is possibly involved in the binding between FPR1/FPRL1 and RAGE. The function of this interaction remains unclear. However, our previous results showed a physical and functional interaction between FPR1/FPRL1 and MARCO [11]. The findings suggest that the receptors interaction influences the receptor activity by cross-phosphorylation and desensitisation of downstream signalling pathways. Such influence was also detected for other receptor classes. For example, studies have shown an extensive cross-talk between opioid- and somatostatin-receptors mediated analgesic responses and pain-processing pathways [43,44]. For the interaction between FPR1/FPRL1 and MARCO as well as RAGE, it is possible that the pattern recognition receptors complement, modulate and enhance each other of their effects. By this, they could enhance the response of the innate immune system or even the inflammatory response in Alzheimer's disease.

For the signal transduction pathways, our results for ERK1/2 phosphorylation and the change of cAMP accumulation show that the specific formyl peptide receptors antagonist WRW4 inhibited RAGE ligands S100B- as well as AGE-BSA-induced glial cell activation (Figure 4). Furthermore, the findings confirmed the importance of the FPRL1 in Aβ1–42-induced signal transduction. However, the results with transfected HEK293 cells showed that S100B- or AGE-BSA-induced ERK1/2 phosphorylation and inhibition of cAMP level is not mediated by FPRL1, whereas FPR1 is involved (Figures 8 and 9). Interestingly, FPR1 expressing HEK293 cells also mediate an Aβ1–42-induced ERK1/2 phosphorylation and a change of cAMP accumulation, whereas our results did not confirm an involvement of RAGE. In addition, a co-transfection of RAGE in FPR1 or FPRL1 expressing cells resulted in an amplification of the S100B- as well as AGE-BSA-induced signal transduction. For the involvement of the receptors in S100B or Aβ1–42-induced signalling, the immunofluorescence and quantitative statistical co-localisation confirmed that RAGE but also FPR1 binding S100B, whereas the co-localisation of Aβ1–42 and FPRL1 is strongest (Figure 10). Altogether, the findings suggest complementary and synergic action between the receptors. The elucidation of consequences for receptor activities and inflammation as well as the progression of the AD need further investigations. Nevertheless, previous kinetics studies showed a binding of Aβ and RAGE in endothelial cells and cortical neurons [45]. I can be assumed that the binding depends on the Aβ forms (monomeric, oligomeric or fibrillary). For the present study, we used non-fibrillary Aβ1-42. In addition, it must be noted that in the primary cells, the mediation of the effect by other receptors cannot be excluded. Furthermore, it was shown that microglial cells behave differently depending upon age in interaction with fibrillary Aβ [46]. Further studies with receptor-deficient cells should bring more clarity here.

In conclusion, there is a substantial interest to identify the cell surface receptors that bind and mediate the intracellular effects of Aβ1-42 in glial cells. Consequently, FPR1/FPRL1 and RAGE or MARCO interactions may explain how formyl peptide receptors interact with a menagerie of structurally diverse pro- and anti-inflammatory ligands associated with different diseases including amyloidosis, Alzheimer’s disease, prion disease and HIV, or with bacterial components [14,21]. Interactions with other receptors may support and modulate the cellular reaction to such structurally diverse ligands by the formyl peptide receptors. Altogether, we hypothesise that formyl peptide receptors play a central role in neurodegenerative mechanisms and physiological regulatory processes.

## Abbreviations

Aβ: Amyloid-β-peptide; DMEM: Dulbecco's modified Eagle's medium; ERK: Extracellular signal-regulated kinases; FCS: Fetal calf serum; fMLF: Formyl-methionyl-leucyl-proline; FPRL1: Formyl peptide receptor like-1; GFAP: Glial fibrillary acidic protein; MARCO: Macrophage receptor with collagenous structure; RAGE: Receptor for advanced glycation endproducts.

## Competing interests

The authors declare that they have no competing interests.

## Authors’ contributions

AS, JM and LOB designed as well as performed experiments, and drafted the manuscript. AE and FM performed experiments. SJ helped to accomplish experiments and revised the manuscript. CJW and TP co-conceived of the study, participated in its design and coordination, and helped draft the manuscript. All authors have read and approved the final version of this manuscript.
